# Supplementary figures and images for: Generally rare but occasionally severe weight gain after switching to an integrase inhibitor in virally suppressed AGEhIV cohort participants
Source: PLoS One. 2021 May 5;16(5):e0251205. doi: 10.1371/journal.pone.0251205 (PMC8099065; doi:10.1371/journal.pone.0251205)

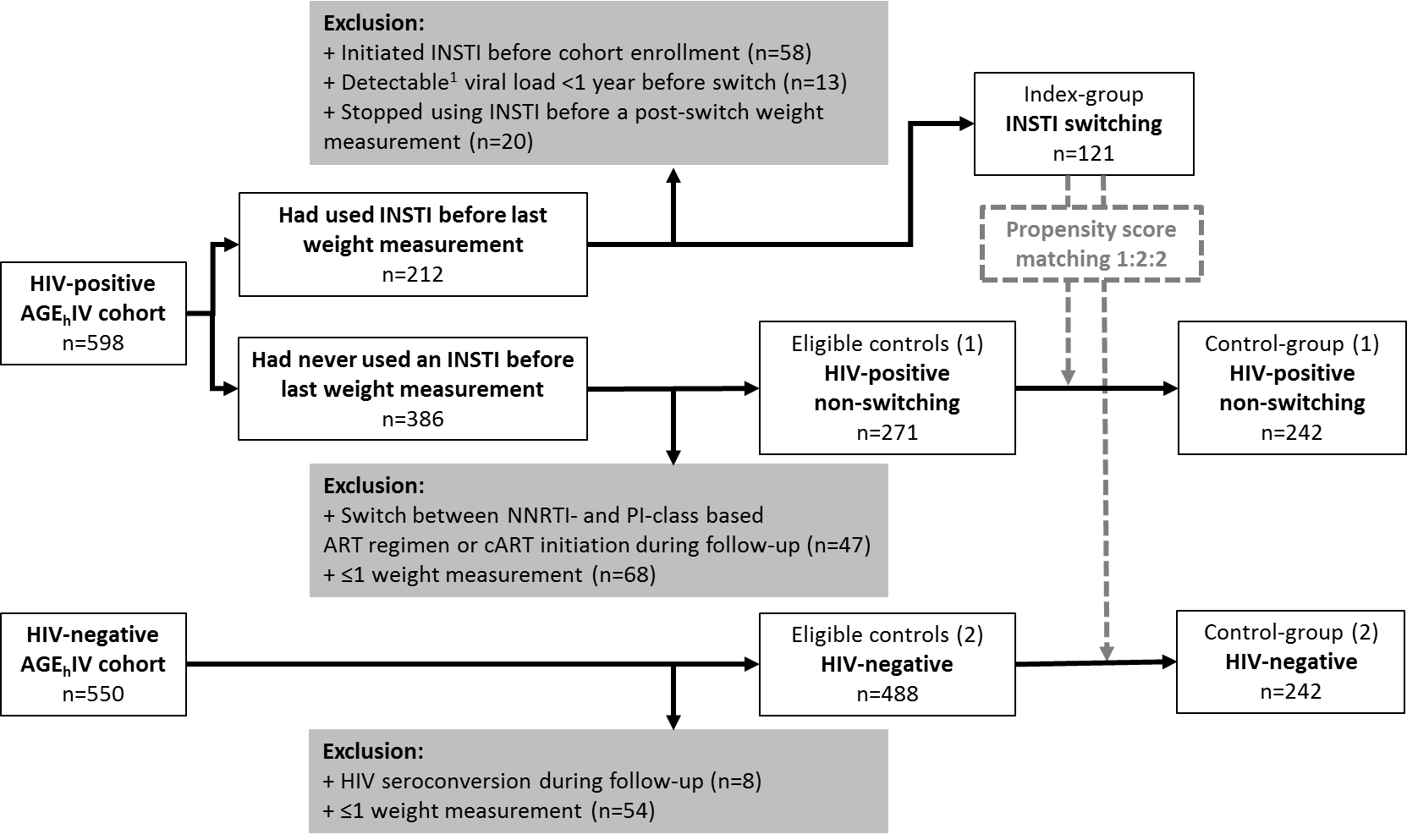

Supplement: S1 Fig — 1HIV viral load >40 copies/mL excluding ‘blips’ up to 200 copies/mL. Abbreviations: INSTI, integrase strand transfer inhibitor; NNRTI, non-nucleoside reverse transcriptase inhibitor; cART, combination antiretroviral therapy; PI, protease inhibitor. (TIF) [file pone.0251205.s001.tif]

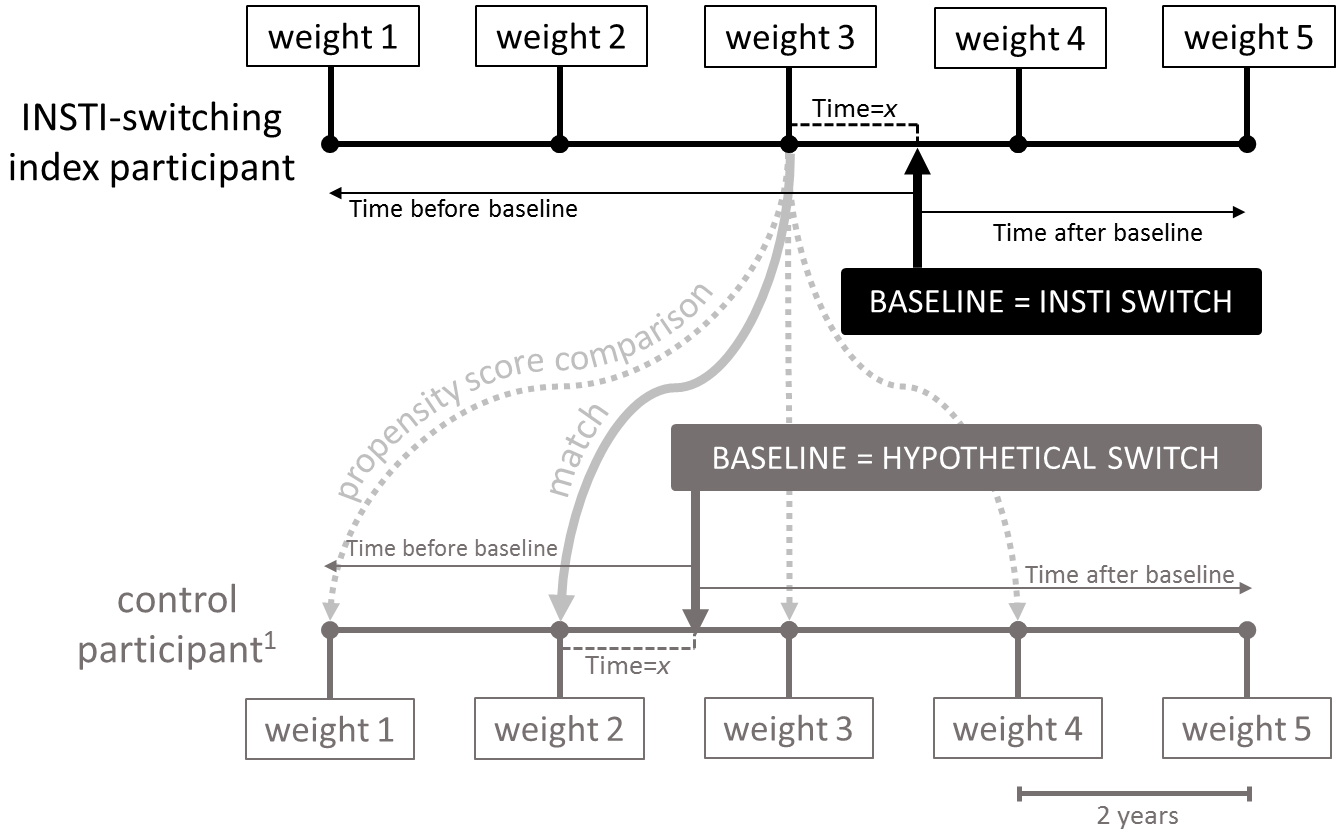

Supplement: S2 Fig — 1HIV-positive non-switching or HIV-negative control. Propensity scores were calculated using a Cox proportional hazard model, including time-updated age and body mass index, and time-fixed gender and ethnicity. Abbreviations: INSTI, integrase strand transfer inhibitor. (TIF) [file pone.0251205.s002.tif]
