# Supplementary material for: Generally rare but occasionally severe weight gain after switching to an integrase inhibitor in virally suppressed AGEhIV cohort participants
Source: PLoS One. 2021 May 5;16(5):e0251205. doi: 10.1371/journal.pone.0251205 (PMC8099065; doi:10.1371/journal.pone.0251205)
Supplement: S1 Table — (DOCX) [file pone.0251205.s003.docx]

**S1 Table. Nucleos(t)ide reverse transcriptase inhibitor use before and after switch to INSTI**

|  | | NRTI use **after switch** to INSTI | | | | | | |
| --- | --- | --- | --- | --- | --- | --- | --- | --- |
|  |  | ABC/3TC | TDF | TDF/FTC | 3TC | 3TC/AZT | TAF/FTC | no NRTI |
| NRTI use **before switch** to INSTI | ABC/3TC | 20 (17%) | … | …. | 1 (1%) | … | 3 (2%) | 1 (1%) |
|  | ABC/3TC/AZT | 3 (2%) | … | 1 (1%) | … | … | … | 1 (1%) |
|  | TDF | … | 1 (1%) | … | … | … | … | … |
|  | TDF/ABC/3TC | … | 1 (1%) | … | … | … | … | … |
|  | TDF/FTC | 14 (12%) | … | 38 (32%) | 3 (2%) | 1 (1%) | 21 (17%) | 3 (2%) |
|  | TDF/3TC | 2 (2%) | … | 1 (1%) | … | … | … | … |
|  | 3TC/AZT | 1 (1%) | … | … | 1 (1%) | 1 (1%) | 1 (1%) | 1 (1%) |
|  | no NRTI | … | … | … | … | … | … | 1 (1%) |

Numbers are n (%). Abbreviations: ABC, abacavir; 3TC, lamivudine; TDF, tenofovir disoproxil; FTC, emtricitabine; AZT, zidovudine; TAF, tenofovir alafenamide; NRTI, nucleoside reverse transcriptase inhibitor; INSTI, integrase strand transfer inhibitor.
