# Supplementary material for: Generally rare but occasionally severe weight gain after switching to an integrase inhibitor in virally suppressed AGEhIV cohort participants
Source: PLoS One. 2021 May 5;16(5):e0251205. doi: 10.1371/journal.pone.0251205 (PMC8099065; doi:10.1371/journal.pone.0251205)
Supplement: S2 Table — (DOCX) [file pone.0251205.s004.docx]

**S2 Table. Primary reasons for switching to INSTI**

|  | N (%) |
| --- | --- |
| Simplification of regimen | 41 (34%) |
| Neuro-/psychological side-effects from previous regime | 26 (21%) |
| Somatic side-effects from previous regime | 31 (26%)^1^ |
| Interaction of co-medication with previous regime | 9 (7%) |
| Unknown | 14 (12%) |

^1^of which 6 (5% of total) were gastro-intestinal complaints
