# Supplementary material for: Generally rare but occasionally severe weight gain after switching to an integrase inhibitor in virally suppressed AGEhIV cohort participants
Source: PLoS One. 2021 May 5;16(5):e0251205. doi: 10.1371/journal.pone.0251205 (PMC8099065; doi:10.1371/journal.pone.0251205)
Supplement: Attachment — Submitted filename: 20_Weight gain AGEhIV cohort_Review_09.11.2020.docx [file pone.0251205.s005.docx]

First of all I would like to mention that the work done is good, very methodical and correct. In general, it seems to me that the objectives of the paper today cover a very interesting and tendentious topic.

**Major comments:**

I believe my most important comment is about the population evaluated in this study. I think that the authors explain and justify very well the fact that women and population with African ethnicity are underrepresented in their study, but they do not give relevance and forget to mention the role of weight gain in the population that does represent them, the AGEhIV cohort. I think that the role that age can have as an independent factor in weight gain is not considered and I think that this could have a potential exploitation given the anthropomorphic changes that the aging population experiences, HIV negative and HIV positive. Especially if the cohort that is being evaluated aims to assess comorbidities in the aging of the HIV population.

**Minor comments:**

- The abstract is excellent, informative and concise.
- The scientific background is well documented, with references of the most important studies up to date. Once again I suggest mentioning age and weight gain relation. I also would recommend including the hypotheses in the introduction.
- In the methods section, the setting and design of the study are well explained, as is the selection of participants and follow-up. The matching criteria are clear. I recommend explaining the potential confounders, and effect modifiers.
- The results are clear; I have not found errors in the tables.
- I really like the discussion. But I repeat for the third and last time, I think it would be interesting in this paper to give relevance to age since it would make this work different from other papers and studies already published.
